# One-stage vs. two-stage bilateral total hip arthroplasty: no difference in clinical outcomes, complications and revision rates in at 5-year follow up

**DOI:** 10.3389/fsurg.2025.1544920

**Published:** 2025-06-05

**Authors:** Alessandra Monzio Compagnoni, Alice Montagna, Giada Accatino, Francesco Benazzo, Mario Mosconi, Michela Saracco, Federico Alberto Grassi, Eugenio Jannelli

**Affiliations:** ^1^ Department of Clinical, Surgical, Diagnostic and Pediatric Sciences, University Study of Pavia, Pavia, Italy; ^2^ U.O.C Orthopedics and Traumatology Poliambulance Foundation, Robotic Prosthetic Surgery Section–Sports Traumatology Unit, Brescia, Italy; ^3^ IRCCS Policlinico San Matteo Foundation, Orthopedics and Traumatology Clinic, Pavia, Italy; ^4^ Department of Orthopaedics and Trauma, “San Giovanni di Dio” Hospital, Naples, Italy

**Keywords:** total hip arthroplasty, THA, one stage, two stage, osteoarthritis abstract

## Abstract

**Background/objectives:**

Total hip arthroplasty (THA) is an effective treatment for end-stage hip disorders, improving pain, function, and quality of life. For bilateral hip disease, choosing between one-stage (simultaneous) and two-stage (staged) bilateral THA is critical. One-stage THA may reduce hospitalization but carries higher risks. This study evaluates whether one-stage bilateral THA is non-inferior to two-stage THA in safety and functional recovery.

**Methods:**

A retrospective study of patients undergoing bilateral THA between January 2010 and November 2019 compared one-stage (84 patients) and two-stage (63 patients) procedures. Surgeries used a postero-lateral approach with H-Max and DeltaTT implants. Outcomes included pain (VAS), function (Oxford Hip Score, EQ-5D, Forgotten Joint Score), and complications. Follow-ups were conducted at 1, 3, and 6 months, and at 5 years for both groups.

**Results:**

Both groups showed significant improvements in pain and function scores with no differences between them. Forgotten Joint Score was also comparable. Complications, including urinary infections and hematomas, were similar, and no revisions occurred in the one-stage group, compared to a 1.6% revision rate in the two-stage group. Hemoglobin decrease and transfusion rates were comparable.

**Conclusions:**

Both approaches resulted in improved symptoms and quality of life with similar complication rates. The one-stage group had fewer complications and no revisions, suggesting potential cost savings. These findings support the safety of one-stage bilateral THA, emphasizing the need for careful patient selection and surgical expertise.

## 1 Introduction

Total hip arthroplasty (THA) has revolutionized the management of end-stage hip disorders and is recognized as one of the most impactful and successful orthopedic procedures performed in the last half-century (1). This surgical intervention is highly effective in alleviating pain, restoring joint function, and significantly improving patients’ quality of life and ability to perform daily activities (2). Over the years, advances in surgical techniques, prosthetic designs, and perioperative care have further contributed to the procedure's success, resulting in high levels of patient satisfaction and durable outcomes (3).

The primary indication for THA is end-stage hip osteoarthritis, which is a leading cause of disability worldwide (4). For many patients, unilateral THA addresses the primary source of their symptoms; however, studies suggest that a substantial proportion of these individuals may develop contralateral hip degeneration, necessitating a second procedure within 10 years (5). In addition to osteoarthritis, various other conditions, such as avascular necrosis, rheumatoid arthritis, ankylosing spondylitis, and developmental dysplasia, can lead to bilateral hip involvement (6). When both hips are affected, the decision to perform bilateral THA becomes a critical consideration for optimizing patient outcomes (7).

In cases of bilateral hip disease, surgeons must decide between two approaches: one-stage (simultaneous) bilateral THA, where both hips are replaced during a single operation, and two-stage (staged) bilateral THA, where each hip is addressed in separate procedures (8). While simultaneous bilateral THA can potentially reduce the overall burden of surgery by requiring a single anesthetic and hospitalization, the decision is not straightforward, as the approach carries unique risks and considerations (9).

Since Charnley et al. first introduced simultaneous bilateral THA in 1971, researchers have sought to understand the relative advantages and disadvantages of these two approaches (10, 11). Both strategies have demonstrated benefits, but controversy remains regarding their comparative safety and efficacy (12). While some evidence suggests that one-stage bilateral THA may be associated with comparable complication rates to unilateral THA, other studies indicate that it may carry higher risks, including greater transfusion rates, increased adverse events, and suboptimal recovery (9). Despite these debates, simultaneous bilateral THA remains an attractive option for certain patient populations, particularly when minimizing hospital stays and recovery periods is *a priori*ty (13).

In this context, the aim of the present study is to determine whether one-stage bilateral THA is noninferior to two-stage bilateral THA in terms of safety and functional recovery. Through this approach, we aim to address important questions regarding the optimal surgical strategy for managing bilateral hip disease and provide valuable evidence to inform clinical decision-making.

## 2 Materials and methods

This retrospective study analyzes patients who underwent bilateral total hip replacement surgery between January 2010 and November 2019. It compares two groups: one cohort that underwent onestage bilateral surgery and a control cohort that underwent two-stage bilateral surgery, with the two procedures occurring within 12 months. Data were gathered using the hospital's computerized record system.

### 2.1 Population

**Figure 1:**
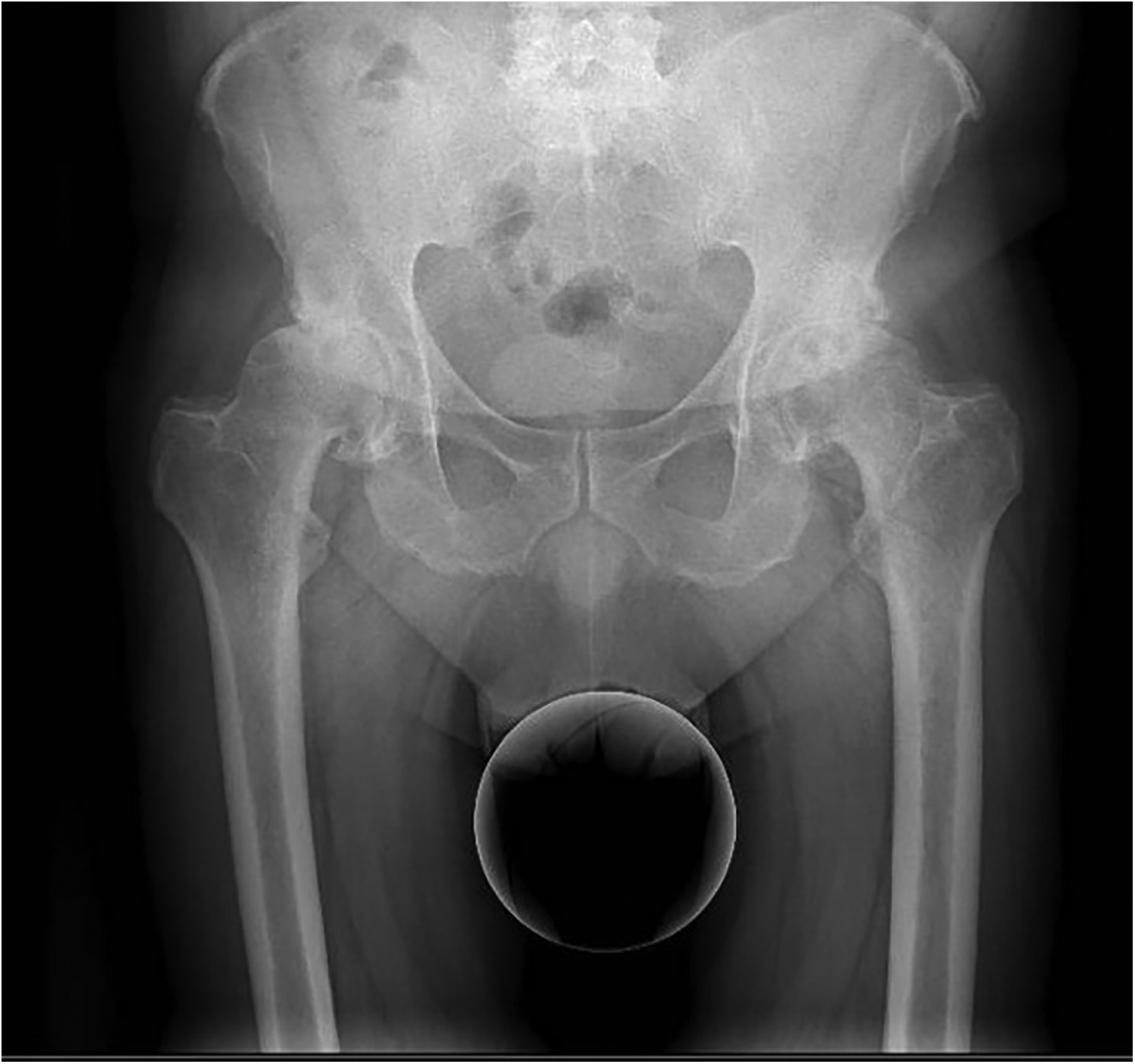
Severe bilateral osteoarthritis of the hip.

The inclusion criteria were as follows: all patients enrolled in the study had a definitive indication for bilateral surgical treatment from the outset, with a Tönnis classification score of 2 or 3 (Figure 1). All patients of the staged group present bilateral symptoms e radiological signs at the time of the diagnosis.

Notably, no patients had previously undergone hip replacement surgeries or conservative surgical procedures. Exclusion criteria included a history of fractures, secondary arthritis resulting from systemic disorders, or femoral head necrosis. At the moment of the diagnosis, the criteria for selecting the patients for one-stage or two-stage total hip arthroplasty (THA) were based on clinical factors: the presence of very complex bilateral coxarthrosis led to the choice of a two-stage operation. Patient preferences and preoperative anesthetic evaluation were also considered. The patients in the staged group are similar to those in the simultaneous group in terms of disease severity, comorbidities, and clinical symptoms.

### 2.2 Surgical technique and post-operative protocol

A single surgeon performed all the surgeries. Each procedure was carried out through a posterolateral approach, with the patient positioned in lateral decubitus. Blocking supports were placed at the sacral level and on the antero-superior iliac spine to ensure stability during the procedure. The capsular incision was made in a T-shape, and the capsule was reattached whenever possible. The rotator muscles were also reinserted to preserve joint function. Notably, none of the prostheses used in the procedures required a cemented stem.

All implants utilized in the surgeries were H-Max for the stem and DeltaTT for the acetabular component (LimaCorporate, Udine, Italy).

The average surgical time for the one-stage procedure was 55 min per side, with a range of 45–90 min. In contrast, the surgical time for prostheses implanted one side at a time was 50, with a range of 45–65. This time was measured from the preparation of the surgical field to the completion of suturing and application of the dressing.

**Figure 2:**
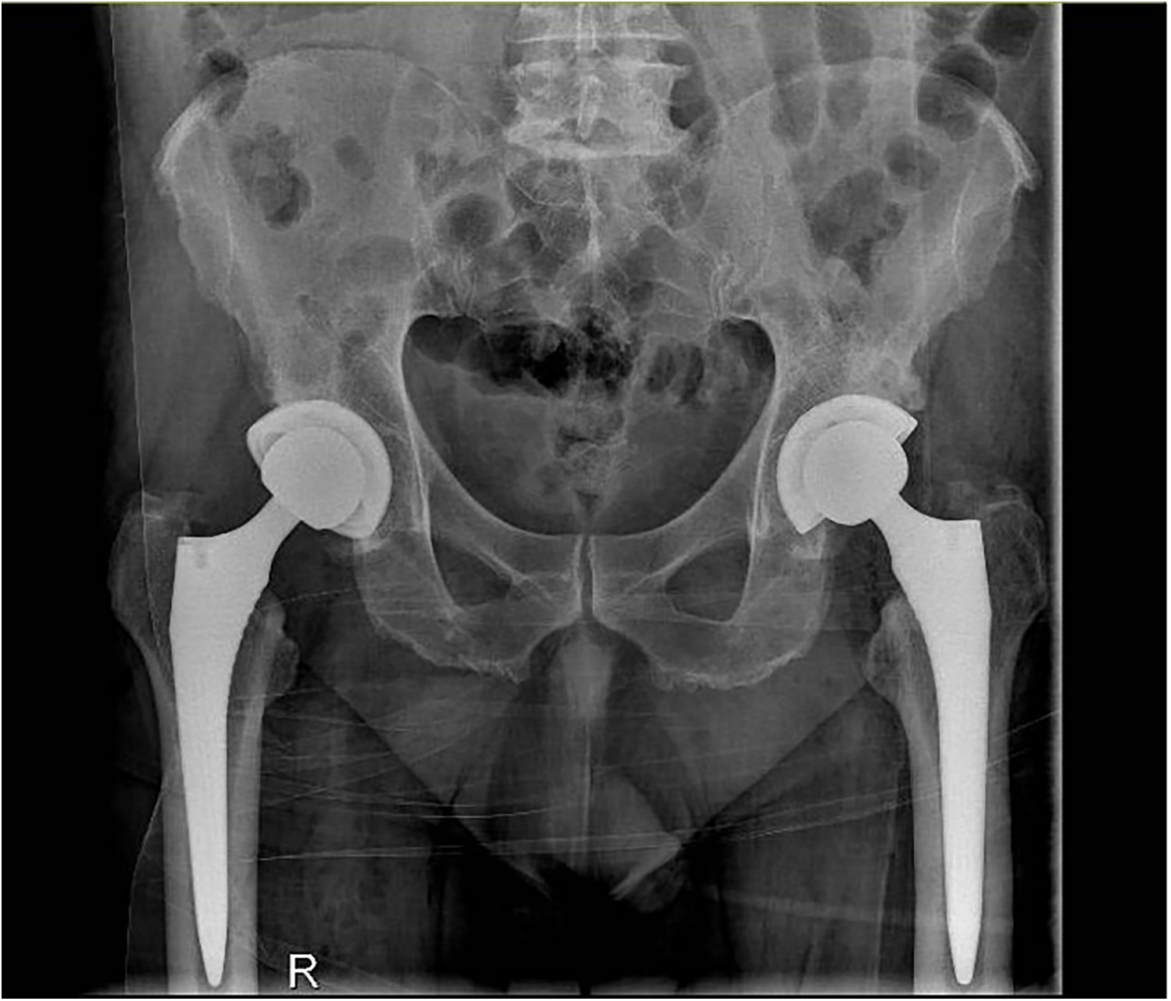
Postoperative radiography of bilateral total hip arthroplasty.

Antibiotic prophylaxis with cefazolin was administered to all patients. Postoperative radiographs were performed immediately after surgery (Figure 2) to assess the positioning of the implants and ensure there were no complications.

According for the surgery of the one sage-procedurs, it is important underlined that after the simultaneous one hip operated on first; the patient was repositioning for the second operative tim. Both procedures were performed under the same anesthesia (general or spinal). Two different and separates sets of surgical instruments were been used for each hip.

The type of rehabilitation and mobilization was the same for the two groups. Additionally, all patients were equipped with anti-dislocation devices (wedge cushion or abduction pillow between knees for sleeping, toilet seat raiser or seat raiser) apartand received detailed guidance on positions to avoid, particularly to mitigate the risk of dislocation associated with the postero-lateral surgical approach.

### 2.3 Follow-up

All patients were regularly followed up at our clinic at 1 month, 3 months, and 6 months after surgery for the case group and after the final surgery for the control group. The final evaluation was conducted at 5 years.

The primary parameter assessed was pain, evaluated both pre- and postoperatively using the Visual Analogue Scale (VAS). The VAS is a validated, subjective tool for measuring acute and chronic pain, where patients indicate their pain level by marking a point on a continuum between “no pain” and “worst pain.”

To assess patient well-being and satisfaction, we utilized patient-reported outcome measures (PROMs), collecting data through questionnaires administered preoperatively, at 6 months, and at 5 years postoperatively. The Oxford Hip Score (OHS), consisting of 12 questions, evaluates an individual's functional ability, activities of daily living, and the impact of pain over the previous four weeks (14). The European Quality of Life EQ-5D is a widely used, standardized measure of healthrelated quality of life across five dimensions: mobility, self-care, usual activities, pain/discomfort, and anxiety/depression (15). The Forgotten Joint Score (FJS) comprises 12 items assessing the extent to which patients can forget the presence of their artificial joint during daily activities (16).

During interim follow-ups, patients were asked about postoperative complications, including allergies, adverse events, urinary issues, bleeding, or wound dehiscence. Perioperative variations in hemoglobin levels and the need for blood transfusions were also recorded. This information was obtained from the computerized blood test system and anesthesiology records. Transfusion thresholds were based on Patient Blood Management (PBM) guidelines, with hemoglobin levels below 7 g/dl or below 8 g/dl in symptomatic or cardiac patients as the criteria for transfusion.

Finally, any adverse events, including the onset of infections, were carefully monitored and documented.

### 2.4 Statistical analysis

The data obtained in this study were analyzed using Microsoft 365 Excel software V2021. The values of the two populations were assessed as homogeneous, so a two-tailed *t*-test was used for independent variables in the case–control comparison, whereas a paired-variables *t*-test was used in the pre- and post- operation comparison. A *p* value <0.05 was considered statistically significant.

### 2.5 Ethical committee approval

All studies were approved by the Lombardia 6 Regional Ethics Committee (approval number: 20150001968), ensuring compliance with ethical standards and the protection of patients’ rights.

## 3 Results

### 3.1 Demographic analysis

Between January 2010 and November 2019, a total of 125 one-stage bilateral hip replacement surgeries were selected. Out of this group, 41 patients were excluded from this study for the following reasons: 33 patients have a femoral head necrosis (due to chemotropic oncologic therapy or cortisonic therapy); 2 patients had coagulopathy (hemophilia); 6 patients were lost to follow-up.

During the same timeframe considered for the one-stage procedures, 110 two-stage bilateral hip replacement surgeries were performed.

Out of the initial group of 110 patients, 44 were excluded from the study for the following reasons: 34 patients presented with femoral head necrosis (attributed to oncologic chemotherapeutic treatments or prolonged corticosteroid therapy); 3 patients had a history of coagulopathy (hemophilia); and 7 patients were lost to follow-up.

The final population consisted of 84 patients undergoing one-stage bilateral hip replacement surgery (case group) and 63 patients undergoing two-stage bilateral hip replacement surgery (control group).

Average time between the two stages was 18 months (with a range of 11–20 months).

The average age for the case group was 71.5 years, with a range of 45–85 years. The gender distribution showed that 34% were male and 66% were female.

The average age for the control group was 72.2 years, with a range of 55–90 years. The gender distribution showed that 44% were male and 56% were female (Table 1).

**Table 1:** Demographic characteristics of the population.

Groups	Case group one-stage THA	Control group two-stage THA	*p* value
No. of patients	84	63	
Age	71.5 [45–85]	72.2 [55–90]	0.08
Gender	M 34% 55 F 66%	M 44% F 56%	0.06
Follow-up [months]	58.8 [12–96]	57.7 [12–80]	0.09

The postoperative follow-up period ranged from a minimum of 12 months to a maximum of 96 months, with an average value of 58.9 months for the case group, whereas the control group followup ranged from a minimum of 12 months to a maximum of 80 months, with an average value of 53.7 months. No statistically significant difference was found in the follow up periods between the two groups (*p* > 0.05).

The case group presented the following comorbidities: four patients had diabetes mellitus, twentyfour had arterial hypertension, eight had coronary artery disease, three had anxiety and depression syndrome, two had liver diseases, and one had kidney diseases. In terms of coxalgia before surgery, 4 patients had had painful symptoms for less than 1 year, 22 for a period between 1 and 5 years, 12 for a period between 6 and 10 years, and 5 for a period exceeding 10 years. The control group presented the following comorbidities: four patients had diabetes mellitus, nineteen had arterial hypertension, six had coronary artery disease, three had anxiety and depression syndrome, and two had liver diseases. In terms of coxalgia before surgery, 6 patients had had painful symptoms for less than 1 year, 34 for a period between 1 and 5 years, 12 for a period between 6 and 10 years, and 11 for a period exceeding 10 years (Table 2).

**Table 2:** Comparison of the number of patients affected by comorbidities in the one-stage procedure vs. the two-stage procedure.

Comorbidities	Case group one-stage TKA	Control group two-stage TKA
Diabetes mellitus	4	4
Hypertension	8	19
Coronary artery disease	8	6
Anxious—depressive syndrome	3	3
Liver disease	2	2
Nephropathy	1	0

### 3.2 Results analysis

**Figure 3:**
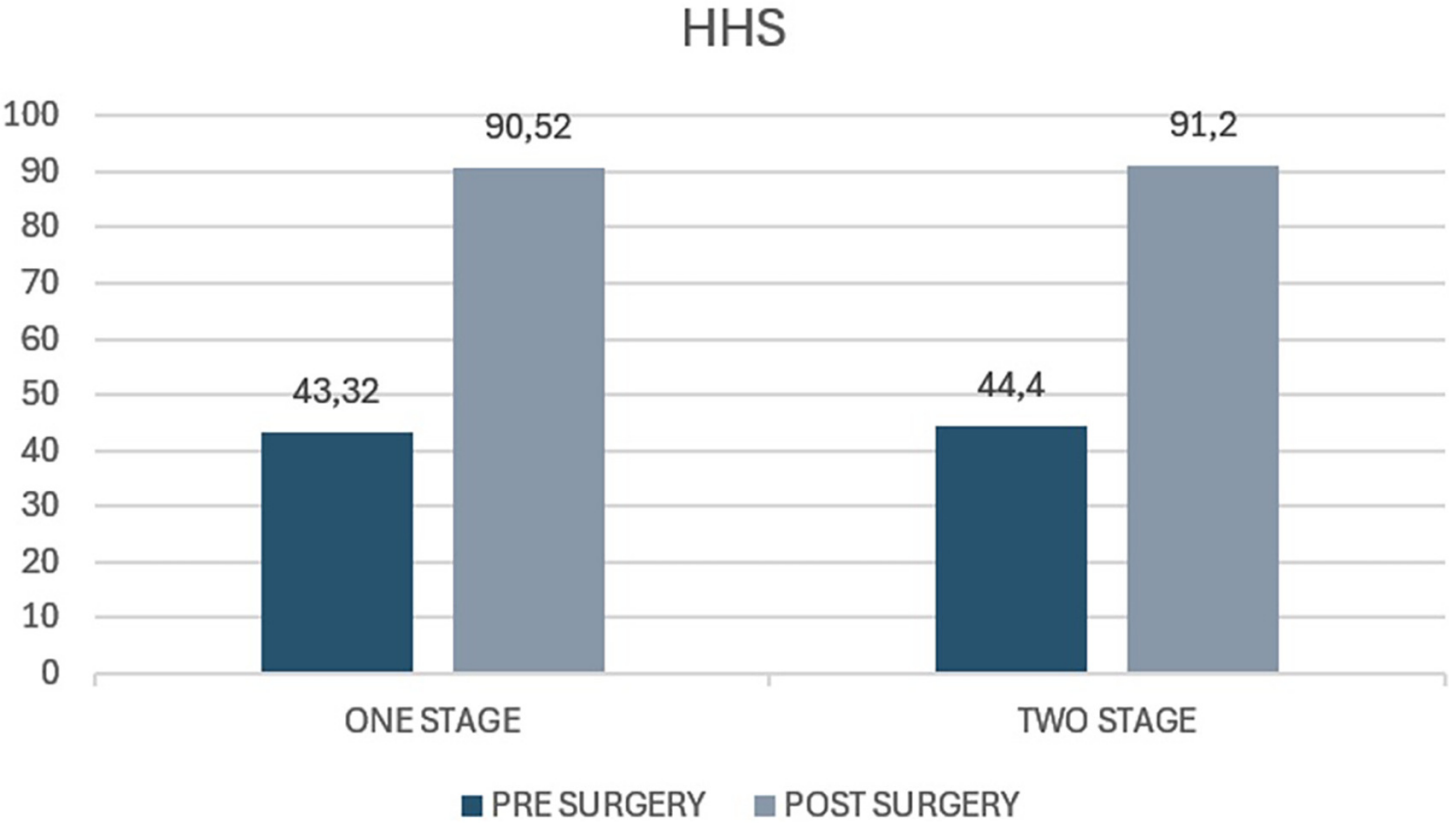
Harris Hip Score (HHS) in patients undergoing one-stage versus two-stage procedures. The figure illustrates preoperative and postoperative HHS values for the “one stage” and “two stage” groups. The y-axis represents the HHS score, ranging from 0 to 100.

The average Harris Hip Score in the case group before surgery was 43.2 (range 32–55.5), whereas the average score at 5 years of follow-up was 90.52 (range 82.5–92). The average increase from preoperative to postoperative values was 47.2 (*p* value <0.05). The average Harris Hip Score before surgery in the control group was 44.4 (range 28.9–53.4) and at the 5-year follow-up, it was 91.2 (range 42–92). The average increase in the score was 46.8 (*p* < 0,05) (Figure 3). Both groups showed a statistically significant improvement following bilateral hip replacement surgery without a statistically significant difference between the two groups (*p* 0.12).

**Figure 4:**
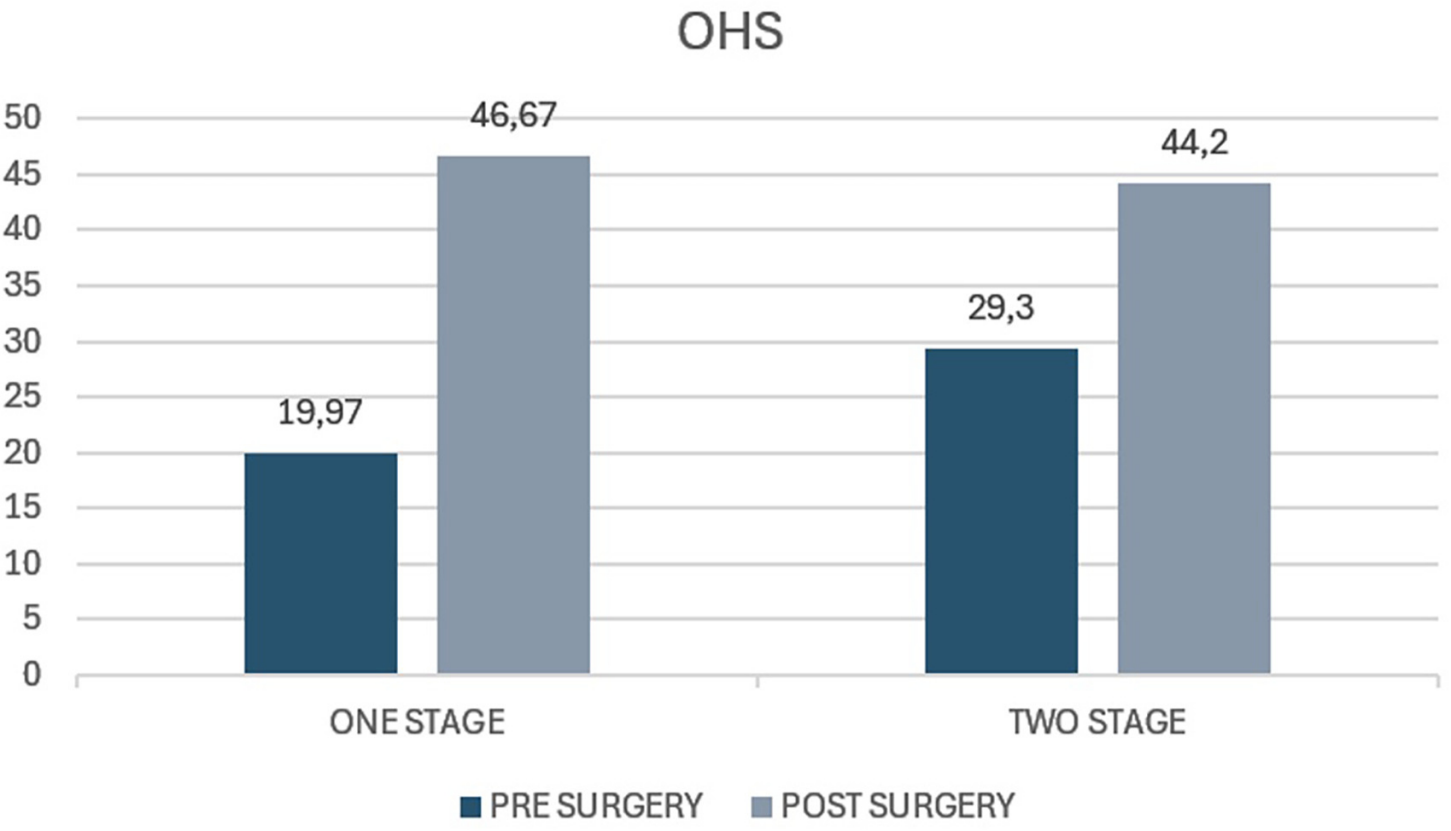
Oxford Hip Score (OHS) in patients undergoing one-stage versus two-stage procedures. The figure presents preoperative and postoperative OHS values for the “one stage” and “two stage” groups. The y-axis represents the OHS score, typically ranging from 0 to 48, with higher scores indicating better hip function.

The average Oxford Hip Score in the case group before surgery was 19.97 (range 12–24.4), whereas the average score at 5 years of follow-up was 46.67 (range 40–48). The average increase from preoperative to postoperative values was 26.7 (*p* < 0.05) (*p* value <0.05). The average Oxford Hip Score before surgery in the control group was 29.3 (range 25–34) and at the 5-year follow-up it was 44.2 (range 34–46). The average increase in the score was 14.9 (*p* < 0.05) (Figure 4). Both groups showed a statistically significant improvement following bilateral hip replacement surgery with no statistically significant difference (*p* value >0.05).

**Figure 5:**
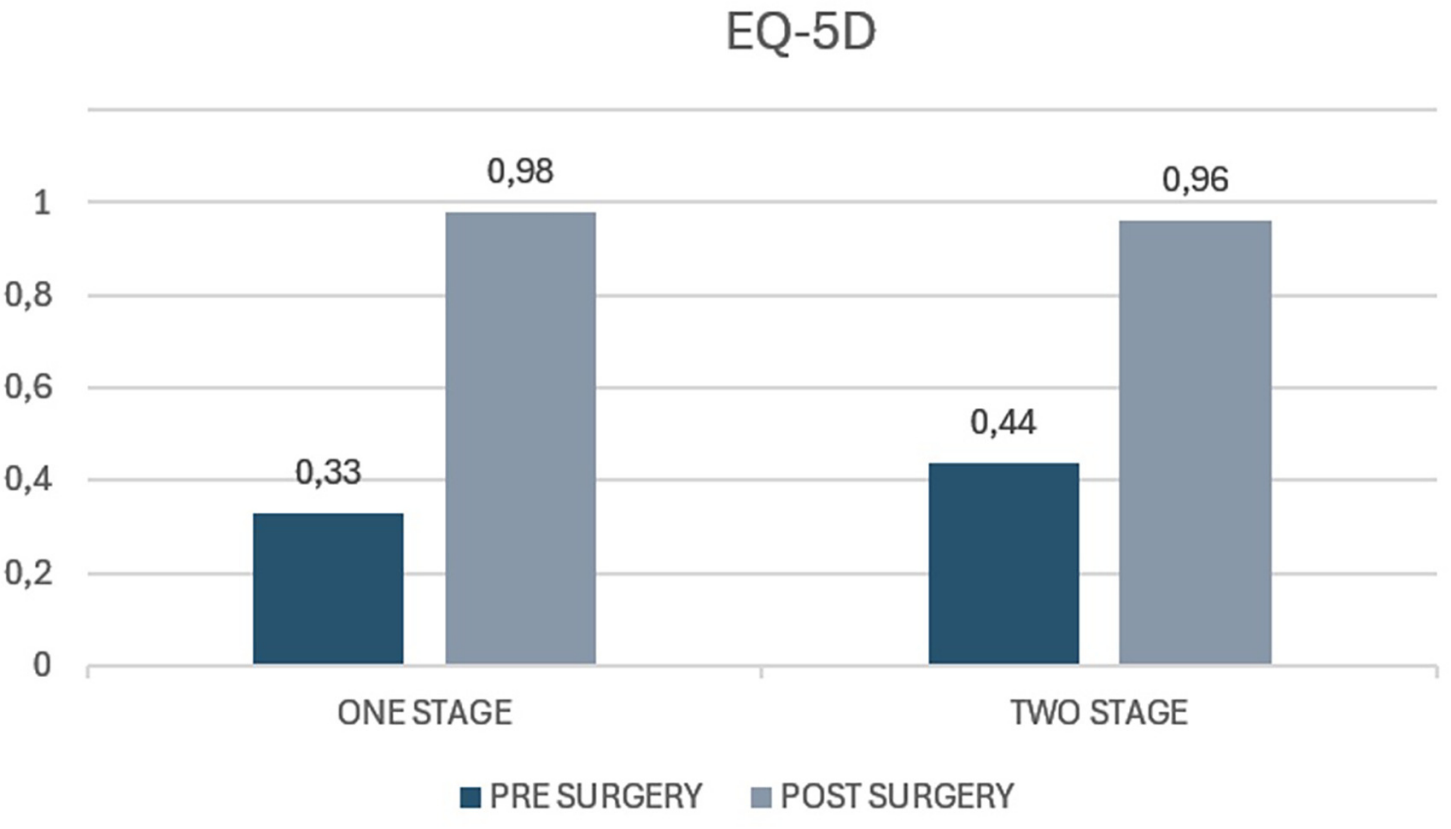
EQ-5D index scores in patients undergoing one-stage versus two-stage procedures. The figure displays preoperative and postoperative EQ-5D index values for the “one stage” and “two stage” groups. The y-axis represents the EQ-5D index score, which typically ranges from 0 (representing the worst health state) to 1 (representing perfect health).

The average EQ-5D value in the case group before surgery was 0.33 (range 0.23−0.90) whereas the average value at 5 years of follow-up was 0.98 (range 0.57–1). The average increase from preoperative values to postoperative values was 0.65 (*p* < 0.05). The average EQ-5D value preoperatively in the control group was 0.44 (range 0.28–0.80) and at the 5-year follow-up, it was 0.96 (range 0.45–1). The average increase in the score was 0.520 (*p* < 0.05) (Figure 5). Both groups showed a statistically significant improvement in quality of life at 5 years of follow-up, even though the average value of the case group was 0,13 higher than control group (*p* = 0.32), with no statistically significant difference (*p* value >0.05).

**Figure 6:**
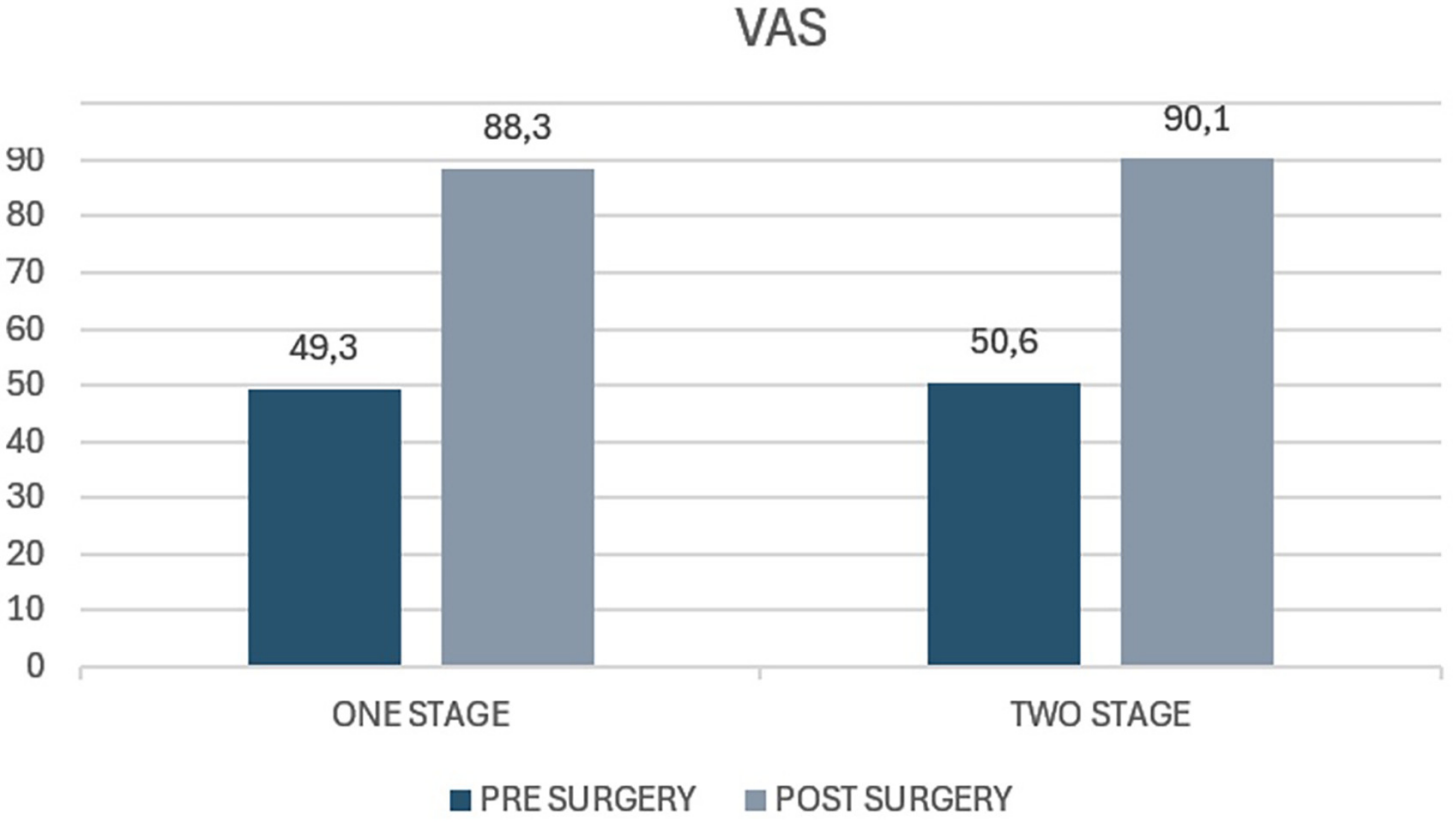
Visual Analog Scale (VAS) pain scores in patients undergoing one-stage versus two-stage procedures. The figure shows preoperative and postoperative VAS scores for the “one stage” and “two stage” groups. The y-axis represents the VAS score, typically ranging from 0 (no pain) to 10 (worst imaginable pain).

The average VAS score in the case group before surgery was 88.3 (range 30–100) whereas the average score at 5 years of follow-up was 49.3 (range 40–60). The average decrease from preoperative to postoperative values was 39 (*p* value <0.05). The average VAS score preoperatively in the control group was 90.1 (range 60–100) and at the 5-year follow-up, it was 50.6 (range 40–60). The average decrease in the score was 39.5 (*p* < 0.05) (Figure 6). The decrease in VAS score in the case group was 0.5 less than in the control group (*p* value = 0.42). No statistically significant difference is reported (*p* 0.09).

**Figure 7:**
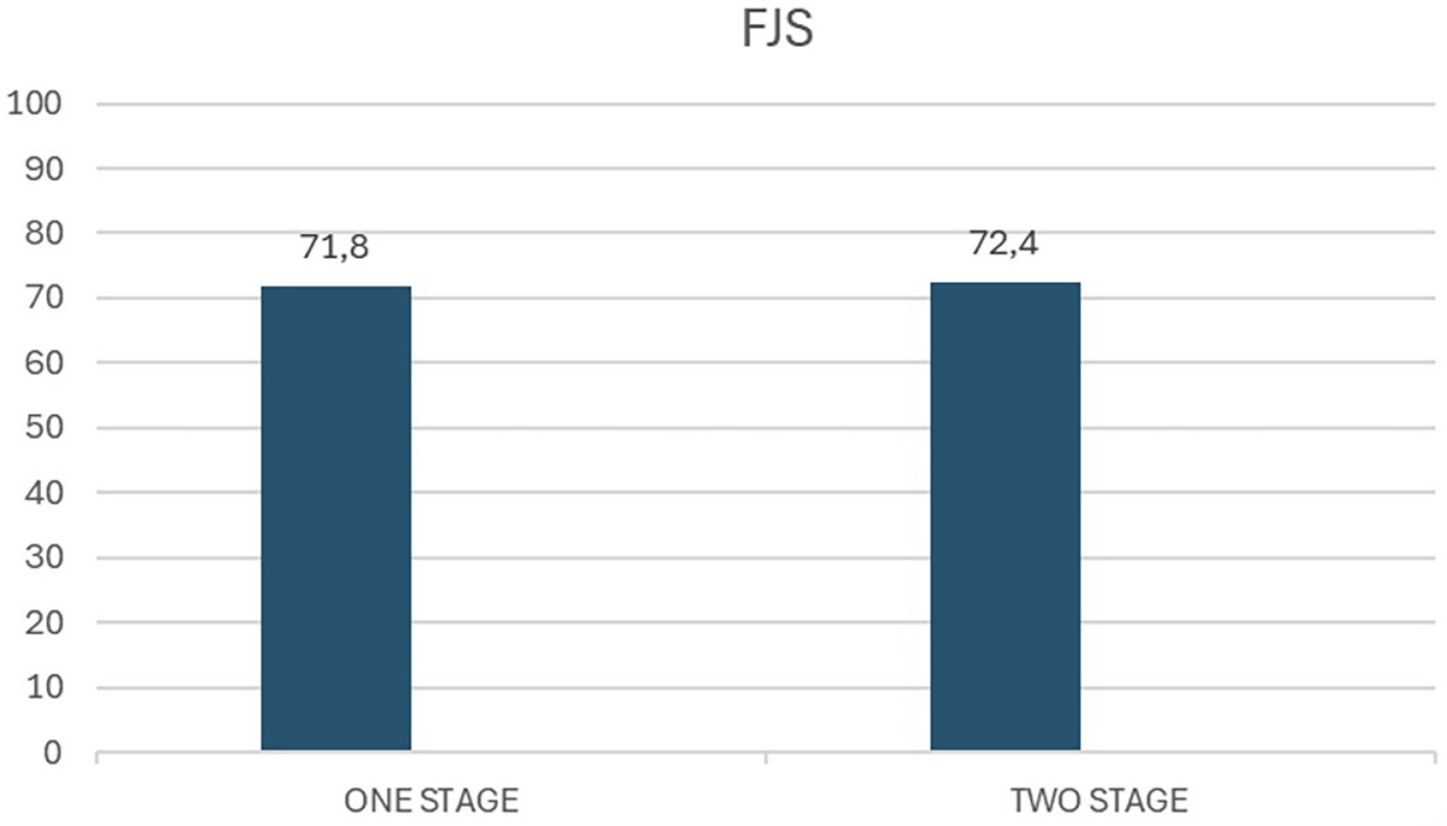
Forgotten Joint Score (FJS) in patients undergoing one-stage versus two-stage procedures. The figure illustrates postoperative FJS values for both the “one stage” and “two stage” groups. The y-axis represents the FJS score, which ranges from 0 to 100, with higher scores indicating a greater ability of the patient to “forget” the joint in daily life (i.e., better joint function and integration).

The average value of the Forgotten Joint Score at 5 years of follow-up in the case group was 71.8 (range 25–100), whereas in the control group it was 72.4 (range 30.4–100). Comparing the two groups, no statistically significant difference was found (*p* value = 0.59) (Figure 7).

### 3.3 Complications

During the postoperative period and intermediate follow-ups, complications were documented, revealing that in the case group 2.4% (2 patients) experienced urinary infections, 2.4% (2 patients) developed hematomas that required a surgical evacuation due to excessive bleeding. One patient had an adverse drug reaction to the peri-operative antibiotic (1.2%). In the control group, 1.6% (1 patient) experienced a urinary infection and 4.8% (3 patients) developed hematomas. About dislocation rates two cases were observed in the one-stage group and one case in the two-stage group. There are therefore no statistically significant differences. The all dislocations occurred within 2 months from the surgery and they were associated with incorrect movements (intrarotation of the hip and iperflexion beyond 90 degrees of the hip, movements described by the same patients).

### 3.4 Revision rates

No revision surgeries were necessary in the case group (0%). In the control group, however, one patient (1.6%) underwent surgical revision for aseptic loosening three years after the initial procedure, which necessitated a one-stage revision surgery.

### 3.5 Hemoglobin

The average decrease in hemoglobin levels per patient was 3.8 g/dl (range 1.6–6.8 g/dl) in the case group. Transfusion of a bag of leukodepleted packed red blood cells was required for 20.9% (9 out of 43) of hospitalized patients. None of the patients required more than one dose of transfusion.

The average decrease in hemoglobin levels per patient was 3.56 g/dl (range 1.4–6.5 g/dl) in the control group. Transfusion of a bag of leukodepleted packed red blood cells was required for 12.4% (12 out of 66) of hospitalized patients. None of the patients required more than one bag of transfusion.

The difference in the mean of transfusion of the two groups was not statistically significant (*p* value =0.27).

### 3.6 Hospitalization and cost

The average hospitalization for patients undergoing two-stage surgery is 4 days for each side, while patients undergoing one-stage surgery have an average hospitalization of 5 days. The one stage intervention probably reduces, overall, hospitalization costs. The length of stay in hospital varied greatly depending on whether patients were discharged home or to a protected rehabilitation; for this reason, no statistical evaluations were carried out.

## 4 Discussion

The primary objective of the present study was to compare the outcomes of bilateral total hip arthroplasty performed in a single-stage procedure vs. a two-stage procedure. The most notable finding of this study is the absence of statistically significant differences in clinical outcomes, necessity of blood transfusions, complications and revision rate between the single-stage and twostage bilateral total hip arthroplasty groups. This result highlights the potential of the single-stage procedure as a viable alternative to the traditional two-stage approach, offering comparable efficacy while potentially reducing recovery time and healthcare resource utilization.

This study boasts a robust follow-up period, with a minimum of 5 years and up to nearly a decade (January 2010 to November 2019), providing valuable long-term insights into the outcomes of onestage and two-stage bilateral total hip arthroplasty.

First of all, it is essential to investigate whether there are differences in complication rates between one-stage and two-stage bilateral total hip arthroplasty to optimize patient outcomes and guide surgical decision-making. In our study we observed a low overall complication rate in both the case and control groups, aligning with previous research that suggests the safety of the single-stage approach. Our findings align with the meta-analysis conducted by Shao et al., which reported that one-stage bilateral THA demonstrated a lower risk of major systemic complications and no significant differences in mortality, pulmonary embolism, cardiovascular complications, or minor surgical complications between the two techniques (17). Moreover, Aghayev et al. reported fewer local and systemic complications in the single-stage bilateral THA group, regardless of the interval between the two surgeries (18). Similarly, Poultsides et al. observed a lower rate of minor complications in the single-stage group, with no significant differences in major complication rates (19). These findings challenge the commonly held belief within the surgical community that single stage procedures carry a higher risk, as recent literature suggests that complication rates are comparable or even lower compared to the two-stage approach.

Specifically, comparing the necessity of blood transfusions is essential to assess whether a one-stage procedure may pose greater risks to the patient as this can significantly impact postoperative recovery and overall outcomes. Our findings showed that in the case group the average decrease in hemoglobin levels was 3.8 g/dl, with 20.9% of patients requiring a single transfusion, while in the control group, the average decrease was 3.56 g/dl, with 12.4% requiring transfusion; however, the difference in transfusion rates between the two groups was not statistically significant (*p* = 0.27). Our findings regarding transfusion requirements align with those of previous studies in literature. In a randomized trial by Taheriazam et al. the cumulative hemoglobin drop and the number of transfused blood units were reported as equivalent between the one-stage and two-stage THA (7). Agarwal et al. in a retrospective study comparing 48 one stage and 56 two-stage bilateral THA reported reduced total blood loss (280 vs. 440 ml), and fewer blood transfusions (1.6 vs. 2.2 units) for the single stage procedures (20).

Moreover, our study reported no revision surgeries in the one-stage group and a 1.6% revision rate in the two-stage group, with one patient requiring revision due to aseptic loosening three years postoperatively. Similarly, Koutserimpas et al. comparing simultaneous and staged bilateral THA using the direct anterior approach (DAA) found no revisions in the simultaneous group, while the staged group had a 6.8% revision rate, including cases of infection, aseptic loosening, and fracture (21). These findings further support the low revision rates associated with the one-stage procedure, aligning with its demonstrated safety and effectiveness.

Given that total hip arthroplasty (THA) has been hailed as the “operation of the century” due to its exceptional outcomes for patients, our functional results were excellent in both groups, with no statistically significant differences observed in the PROMs utilized in this study (22). Micicoi et al. reported no significant differences in functional outcomes between the two approaches (12). A particularly interesting finding by Eggli et al. is that one-stage procedures may offer superior functional outcomes particularly for patients with significantly stiff hips characterized by a preoperative range of motion (ROM) below 50° (23). By addressing both hips simultaneously in a single-stage procedure, the overall rehabilitation process may be streamlined, allowing for symmetrical recovery and potentially minimizing compensatory gait patterns. Accordingly, Schiessel et al. documented that the patients’ preference is for one-stage procedures since it involves surgical intervention and physiotherapy done once for both hips (24).

Even when compared to unilateral total hip arthroplasty (U-THA), one-stage bilateral THA demonstrates excellent outcomes, as evidenced by one study by Micicoi et al. reporting comparable complication rates, implant survival, and functional results (25). Similarly, our findings confirm that one-stage bilateral THA provides high functional scores and low complication rates, reinforcing its safety and efficacy.

In conclusion, this study highlights the strong clinical outcomes, comparable safety profiles, and absence of increased revision rates in one-stage vs. two-stage bilateral THA, emphasizing its viability as a treatment option. A key strength of this research is the long-term follow-up, exceeding five years, which provides robust evidence for the durability and effectiveness of both approaches, ensuring its relevance for guiding clinical decision-making.

### 4.1 Study limitations

This study has some limitations. First of all, the data and the PROMs were collected retrospectively. Secondly, there is not an accurate standardization of the two groups based on age criteria and anaesthesiologic risks. Another limitation is that the postoperative rehabilitation program developed for each patient's cohort was not considered. Radiographic evaluation during follow-up would have been helpful, but this study was based on clinical evaluations and patient-reported outcome measures. Finally, our results are based on a relatively small cohort of patients, so the reproducibility and the reliability of the procedures should be validated involving a larger study population.

## 5 Conclusions

The study demonstrates significant improvements in symptoms and quality of life for patients in both groups, with notable gains in functional scores. While no definitive differences emerged between the one-stage and two-stage procedures, the complication profiles were comparable. Notably, the onestage group experienced fewer complications overall, including the absence of revision surgeries, compared to the two-stage group.

An important advantage of the one-stage approach lies in its potential to reduce healthcare costs by consolidating treatment into a single hospitalization. Our findings align with existing literature, further supporting the safety of the one-stage procedure. However, this approach demands careful consideration of the patient's health status and the surgical team's expertise to ensure optimal outcomes.

## Data Availability

The data analyzed in this study is subject to the following licenses/restrictions: The dataset generated and analyzed during the current study is not publicly available due to patient confidentiality and privacy restrictions but is available from the corresponding author on reasonable request. Requests to access these datasets should be directed to Alessandra Monzio Compagnoni, alessandramonzio.c@gmail.com.

## References

[1] Harris WH. The first 50 years of total hip arthroplasty: lessons learned. Clin Orthop Relat Res. (2009) 467(1):28–31. 10.1007/s11999-008-0467-1 18982399 PMC2601012

[2] Gould VC Blom AW Wylde V. Long-term patient-reported outcomes after total hip replacement: comparison to the general population. Hip Int. (2012) 22(2):160–5. 10.5301/HIP.2012.9230 22547380

[3] Scott CEH Clement ND Davis ET Haddad FS. Modern total hip arthroplasty: peak of perfection or room for improvement? Bone Joint J. (2022) 104-B(2):189–92. 10.1302/0301-620X.104B2.BJJ-2022-0007 35094584

[4] Parvizi J Ganz R. Hip osteoarthritis. Orthopedics. (2003) 26(11):1099–1109. 10.3928/0147-7447-20031101-04 14627102

[5] Amstutz HC Le Duff MJ. The natural history of osteoarthritis: what happens to the other hip? Clin Orthop Relat Res. (2016) 474(8):1802–9. 10.1007/s11999-016-4888-y 27172820 PMC4925421

[6] Eberhardt K Fex E Johnsson K Geborek P. Hip involvement in early rheumatoid arthritis. Ann Rheum Dis. (1995) 54(1):45–8. 10.1136/ard.54.1.45 7880121 PMC1005511

[7] Taheriazam A Mohseni G Esmailiejah AA Safdari F Abrishamkarzadeh H. Bilateral total hip arthroplasty: one-stage versus two-stage procedure. Hip Int. (2019) 29(2):141–6. 10.1177/1120700018773427 29756496

[8] Trojani C d’Ollonne T Saragaglia D Vielpeau C Carles M Prudhon JL One-stage bilateral total hip arthroplasty: functional outcomes and complications in 112 patients. Orthop Traumatol Surg Res. (2012) 98(6 Suppl):S120–123. 10.1016/j.otsr.2012.06.008 22939864

[9] Muskus M Rojas J Gutiérrez C Guio J Bonilla G Llinás A. Bilateral hip arthroplasty: when is it safe to operate the second hip? A systematic review. Biomed Res Int. (2018) 2018:3150349. 10.1155/2018/3150349 29682533 PMC5851297

[10] Jaffe WL Charnley J. Bilateral Charnley low-friction arthroplasty as a single operative procedure. A report of fifty cases. Bull Hosp Joint Dis. (1971) 32(2):198–214. 5128234

[11] Stavrakis AI SooHoo NF Lieberman JR. Bilateral total hip arthroplasty has similar complication rates to unilateral total hip arthroplasty. J Arthroplasty. (2015) 30(7):1211–4. 10.1016/j.arth.2015.02.015 25737389

[12] Micicoi G Bernard de Dompsure R Boileau P Trojani C. Comparative study of bilateral total hip arthroplasty in one or two stages. Orthop Traumatol Surg Res. (2022) 108(6):103359. 10.1016/j.otsr.2022.103359 35781050

[13] Cheng R Mantena Y Chiu YF Kahlenberg CA Figgie MP Driscoll DA. To stage or not to stage? comparison of patient-reported outcomes, complications, and discharge disposition after staged and simultaneous bilateral posterior total hip arthroplasty. J Arthroplasty. (2024) 39(7):1752–7. 10.1016/j.arth.2024.01.011 38216001

[14] Dawson J Fitzpatrick R Carr A Murray D. Questionnaire on the perceptions of patients about total hip replacement. J Bone Joint Surg Br. (1996) 78(2):185–90. 10.1302/0301-620X.78B2.0780185 8666621

[15] EuroQol Group. EuroQol—a new facility for the measurement of health-related quality of life. Health Policy. (1990) 16(3):199–208. 10.1016/0168-8510(90)90421-9 10109801

[16] Behrend H Giesinger K Giesinger JM Kuster MS. The «forgotten joint» as the ultimate goal in joint arthroplasty: validation of a new patient-reported outcome measure. J Arthroplasty. (2012) 27(3):430–6.e1. 10.1016/j.arth.2011.06.035 22000572

[17] Shao H Chen CL Maltenfort MG Restrepo C Rothman RH Chen AF. Bilateral total hip arthroplasty: 1-stage or 2-stage? A meta-analysis. J Arthroplasty. (2017) 32(2):689–95. 10.1016/j.arth.2016.09.022 27776901

[18] Aghayev E Beck A Staub LP Dietrich D Melloh M Orljanski W Simultaneous bilateral hip replacement reveals superior outcome and fewer complications than two-stage procedures: a prospective study including 1819 patients and 5801 follow-ups from a total joint replacement registry. BMC Musculoskelet Disord. (2010) 11:245. 10.1186/1471-2474-11-245 20973941 PMC2987971

[19] Poultsides LA Triantafyllopoulos GK Memtsoudis SG Do HT Alexiades MM Sculco TP. Perioperative morbidity of same-day and staged bilateral total hip arthroplasty. J Arthroplasty. (2017) 32(10):2974–9.e1. 10.1016/j.arth.2017.05.028 28629904

[20] Agarwal S Gupta G Sharma RK. Comparison between single stage and two stage bilateral total hip replacement- our results and review of literature. Acta Orthop Belg. (2016) 82(3):484–90. 29119888

[21] Koutserimpas C Rob E Servien E Lustig S Batailler C. Similar complications and outcomes with simultaneous versus staged bilateral total hip arthroplasty with the direct anterior approach: a comparative study. SICOT J. (2024) 10:31. 10.1051/sicotj/2024028 39177435 PMC11342850

[22] Learmonth ID Young C Rorabeck C. The operation of the century: total hip replacement. Lancet:370(9597):1508–19. 10.1016/S0140-6736(07)60457-7 17964352

[23] Eggli S Huckell CB Ganz R. Bilateral total hip arthroplasty: one stage versus two stage procedure. Clin Orthop Relat Res. (1996) 328:108–18. 10.1097/00003086-199607000-00019 8653943

[24] Schiessel A Brenner M Zweymüller K. Bilateral hip joint replacement as a one-stage or two-stage procedure for dysplastic coxarthritis: a comparative analysis of 30 patients. Z Orthop Ihre Grenzgeb. (2005) 143(6):616–21. 10.1055/s-2005-918185 16380892

[25] Micicoi G de Dompsure RB Micicoi L Tran L Carles M Boileau P One-stage bilateral total hip arthroplasty versus unilateral total hip arthroplasty: a retrospective case-matched study. Orthop Traumatol Surg Res. (2020) 106(3):577–81. 10.1016/j.otsr.2020.02.003 32265170

